# Rupture diaphragmatique droite avec passage total et isolé du foie en intra-thoracique

**Published:** 2011-10-27

**Authors:** Mohamed Turki, Mohamed Hafed Barhoumi, Hassen Hajji, Heithem Chemchik, Bechir M'barek

**Affiliations:** 1 Service de réanimation et soins intensifs, hôpital Ibn Eljazzar, Kairouan, Tunisie; 2 Service de chirurgie générale, hôpital Ibn Eljazzar, Kairouan, Tunisie; 3 Service d'anesthésie réanimation CHU Sahloul, Sousse, Tunisie

**Keywords:** Diaphragme, traumatisme, thorax, Chirurgie, foie, Tunisie

## Abstract

La rupture traumatique de la coupole diaphragmatique droite avec hernie du foie dans le thorax est une lésion rare. Elle est souvent intégrée dans le cadre d'un poly-traumatisme, dont elle est un critère de gravité. Elle expose, précocement ou tardivement, à des complications cardio-pulmonaires par compression. Le diagnostic d'une rupture diaphragmatique droite est difficile à établir. En effet, ses signes cliniques sont peu spécifiques et l'imagerie peut être prise en défaut du faite qu'elle visualise les organes ascensionnés mais plus difficilement la rupture elle-même. La voie d'abord thoracique est souvent préférée du fait des difficultés de l'exposition du diaphragme en présence du foie. Nous rapportons un cas d'une rupture diaphragmatique droite avec passage isolé et total du foie en intra-thoracique diagnostiquée au cinquième jour d'hospitalisation chez un polytraumatisé.

## Introduction

La rupture diaphragmatique droite est rare et survient chez environ 5% à 20% de toutes les lésions diaphragmatiques [1]. L′incidence d'une hernie des organes intra-abdominaux dans la cavité pleurale est faible et n'est observée que dans environ 19% des ruptures droites [1]. L'objectif de ce cas clinique est d’étudier les mécanismes physiopathologiques, les modalités diagnostiques et thérapeutiques de cette complication.

## Observation

Patient B.N. âgé de 35 ans sans antécédents pathologiques notables a été victime d'un accident de la voie public. L'examen trouvait un patient agité avec un Glasgow à 10. L'examen physique révélait l'absence de murmure vésiculaire du côté droit avec écorchure basi-thoracique droite. Le patient était polypnéique, SpO2 était de 92% à l'air ambiant avec un état hémodynamique correct. Le patient a été intubé, ventilé et sédaté. La radiographie thoracique trouvait un hémothorax droit de grande abondance qui a été drainé.

**Figure 1:**
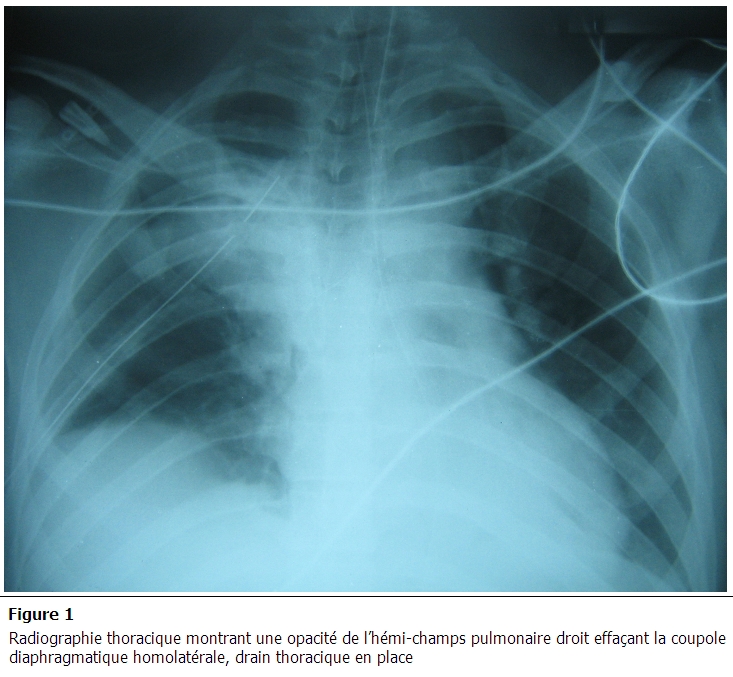
Radiographie thoracique montrant une opacité de l'hémi-champs pulmonaire droit effaçant la coupole diaphragmatique homolatérale, drain thoracique en place

**Figure 2:**
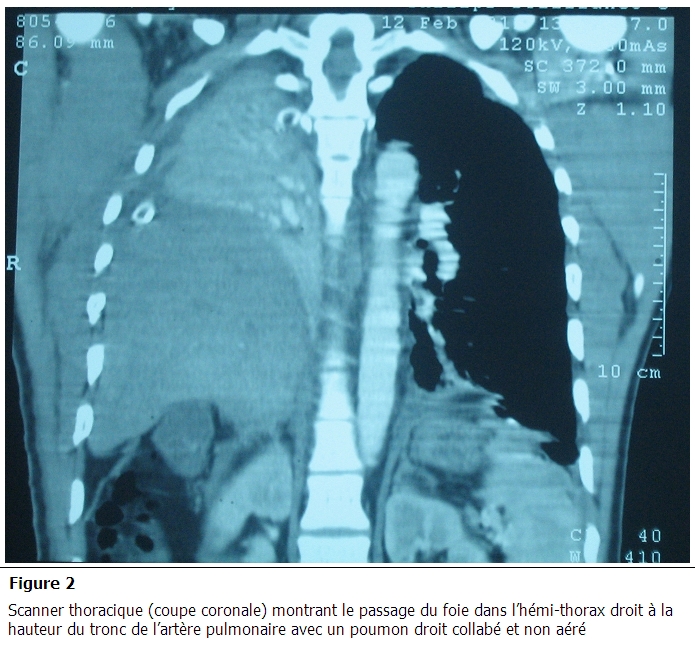
Scanner thoracique (coupe coronale) montrant le passage du foie dans l'hémi-thorax droit à la hauteur du tronc de l'artère pulmonaire avec un poumon droit collabé et non aéré

**Figure 3:**
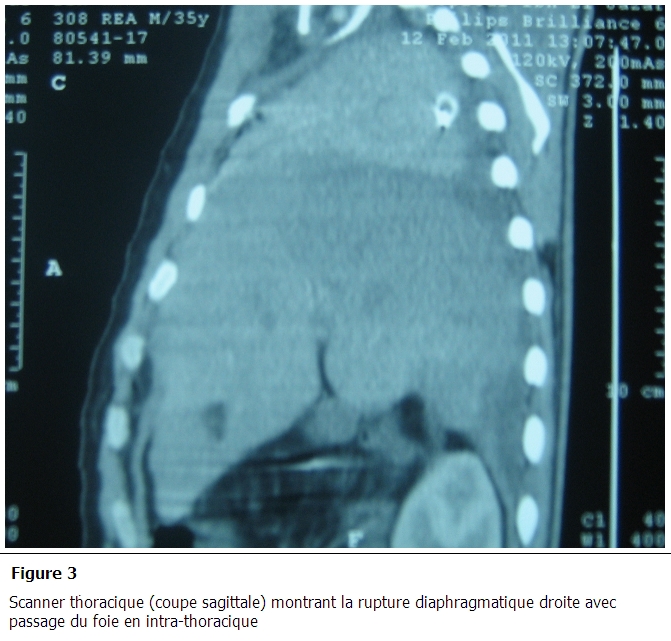
Scanner thoracique (coupe sagittale) montrant la rupture diaphragmatique droite avec passage du foie en intra-thoracique

Un bodyscanner, réalisé après stabilisation du patient, a objectivé de multiples foyers de contusion cérébrale frontaux bilatéraux avec hémorragie méningée de faible abondance, fracture des arcs antérieurs des 1^e^ et 2^e^ côtes droites, hémothorax droit de moyenne abondance, foyer de contusion pulmonaire basal bilatéral avec absence de lésions abdominales associées. Les radiographies faites aux cours de son hospitalisation montraient une opacité de l'hémi-champs pulmonaire droit effaçant la coupole diaphragmatique homolatérale (Figure 1). Le contrôle scanographique réalisé au 5^e^ jour d'hospitalisation montre la stabilité des lésions cérébrales avec disparition de l'hémorragie méningée. Sur le plan thoracique, persistance d'une fine lame d’épanchement pleural liquidien droit et une petite ascension en intra-thoracique du dôme hépatique. La sédation était arrêtée le jour même avec réveil calme, absence de déficit moteur et stabilité de l’état respiratoire. Après six heures de son extubation, le patient présentait une détresse respiratoire avec abolition de murmure vésiculaire du côté droit sans modification de l’état hémodynamique. La radiographie de thorax montre une opacité homogène occupant la quasi-totalité de l'hémi-champs pulmonaire droit. Un scanner thoracique fait en urgence confirme le diagnostic d'une rupture du diaphragme avec hernie du foie dans le thorax en montrant l'ascension du foie dans l'hémi-thorax droit à la hauteur du tronc de l'artère pulmonaire avec un poumon droit collabé et non aéré (Figure 2 et Figure 3).

**Figure 4:**
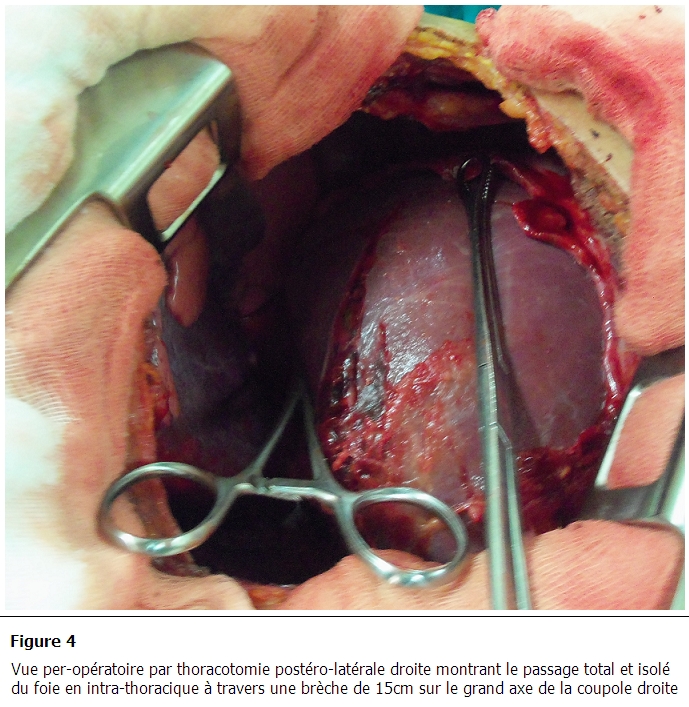
Vue per-opératoire par thoracotomie postéro-latérale droite montrant le passage total et isolé du foie en intra-thoracique à travers une brèche de 15cm sur le grand axe de la coupole droite

Le patient était opéré par une voie d'abord thoracique postéro-latérale droite, avec découverte d'un passage total et isolé du foie en intra-thoracique à travers une brèche de 15cm sur le grand axe de la coupole droite (Figure 4). Le pédicule hépatique était étiré sans rupture décelable. L'intervention consistait en une libération soigneuse des adhérences et en une réintégration du foie en intra-abdominal avec suture de la plaie diaphragmatique par des points séparés au fil résorbable. Les suites opératoires en réanimation étaient marquées essentiellement par un réveil calme après 48H de sédation permettant l'extubation sans incident respiratoire particulier et par l'ablation des deux drains thoraciques successivement à J4 et J7 postopératoire.

## Discussion

Une rupture diaphragmatique doit systématiquement être évoquée lors d'un traumatisme thoracique et/ou abdominal de haute énergie. Le mécanisme de rupture consiste en une élévation brutale de la pression abdominale, jusqu’à dix fois la normale, consécutive à des forces de compression [2]. Les données des séries les plus récentes montrent que les ruptures du diaphragme droit peuvent représenter près de 35% de toutes les lésions diaphragmatiques [3]. Ceci est généralement expliqué par le rôle protecteur de la masse hépatique, et surtout le fait que les ruptures droites sont souvent associées à des lésions vitales graves entraînant le décès avant l'arrivée à l'hôpital [3].

Les principaux risques d'une rupture diaphragmatique droite sont représentés par l'insuffisance de la fonction diaphragmatique, la compression pulmonaire, le déplacement du médiastin et la diminution du retour veineux [4]. En effet, les organes ascensionnés dans le thorax provoquent une élévation paradoxale de la pression veineuse centrale, au même titre qu'une tamponnade ou qu'un pneumothorax compressif.

Le diagnostic préopératoire d'une rupture diaphragmatique est difficile. Vingt à 40% des ruptures sont découvertes lors d'une laparotomie réalisée pour une autre lésion car les signes cliniques sont inconstants et rarement spécifiques [1]. Dans notre cas, le diagnostic n'est retenu qu'après extubation du patient et l'apparition d'une détresse respiratoire inexpliquée. Le passage tardif intra-thoracique du foie s'explique par les modifications brusques du gradient de pression trans-diaphragmatique lors des efforts inspiratoires.

Les moyens diagnostiques comprennent la radiographie pulmonaire, l’échographie, la tomodensitométrie (TDM), l'imagerie par résonance magnétique (IRM). Les radiographies thoraciques ont une sensibilité relativement faible, mais restent un outil de dépistage avec des résultats évocateurs du diagnostic uniquement chez 17 à 40% des patients [5]. La rupture doit être suspectée devant toute élévation marquée de la coupole diaphragmatique avec une hernie intra-thoracique des viscères abdominaux [6]. L′échographie abdominale prolongée au-dessus du diaphragme peut être utile pour le diagnostic. Elle permet d′observer l'absence de mouvements du diaphragme, la hernie des viscères, ou les plans de rupture de membrane [6]. La TDM hélicoïdale est la modalité diagnostique préférée en raison de sa capacité à acquérir des données volumétriques et de bonne qualité des reconstructions coronales et sagittales [7]. La TDM, dans les ruptures diaphragmatiques droites, a une sensibilité de 50 à 90% et une spécificité de 90 à 100% [1,8]. L′IRM, actuellement inutilisable dans le contexte d′urgence, offre des informations identiques à celles du scanner hélicoïdal, mais avec des images directes frontales et sagittales et une meilleure résolution spatiale [9].

En phase aigüe, la voie abdominale est la voie de référence. Elle permet l'exploration et le traitement des viscères abdominaux.

En phase tardive (après 7^e^ jour) et en l'absence de lésions abdominales associées, il semble qu'une thoracotomie droite avec réintégration du foie en intra-abdominal et qu'une réparation par des points séparés au fil non résorbables constituent la stratégie la plus courante devant une rupture diaphragmatique droite. Elle autorise le contrôle d’éventuelles adhérences thoraciques et la mise en place de matériel prothétique, le cas échéant [10].

## Conclusion

La rupture traumatique du diaphragme droit peut entraîner une morbidité et une mortalité importantes. C'est une affection rare, habituellement masquée par de multiples lésions associées, ce qui peut aggraver l'état du patient. Les patients qui sont victimes d'un traumatisme thoraco-abdominale violent devrait y avoir un indice élevé de suspicion de lésion diaphragmatique. Le diagnostic peut être posé par une TDM thoraco-abdominale. La stratégie chirurgicale, au moment du diagnostic, est variable et doit être discutée au cas par cas.
